# In Vitro Antibacterial Activities of Fosfomycin against *Escherichia coli* Isolates from Canine Urinary Tract Infection

**DOI:** 10.3390/ani14131916

**Published:** 2024-06-28

**Authors:** Nattha Jariyapamornkoon, Suphachai Nuanualsuwan, Nipattra Suanpairintr

**Affiliations:** 1 Graduate Program in Veterinary Bioscience, Faculty of Veterinary Science, Chulalongkorn University, Bangkok 10330, Thailand; nattha.j@student.chula.ac.th; 2 Department of Veterinary Public Health, Faculty of Veterinary Sciences, Chulalongkorn University, Bangkok 10330, Thailand; suphachai.n@chula.ac.th; 3 Center of Excellence for Food and Water Risk Analysis (FAWRA), Department of Veterinary Public Health, Faculty of Veterinary Science, Chulalongkorn University, Bangkok 10330, Thailand; 4 Department of Pharmacology, Faculty of Veterinary Science, Chulalongkorn University, Bangkok 10330, Thailand

**Keywords:** dogs, *Escherichia coli*, fosfomycin, minimum inhibitory concentration (MIC), mutant prevention concentration (MPC), urinary tract infection (UTI)

## Abstract

**Simple Summary:**

Canine urinary tract infections (UTIs) caused by *Escherichia coli* (*E. coli*) are common in dogs. While beta-lactams and trimethoprim-sulfonamides are typically the first-line treatments for UTIs, some multidrug-resistant (MDR) *E. coli* strains are resistant to these drugs as well as to second-line treatments like fluoroquinolones. Critically important antibiotics such as carbapenems are reserved for life-threatening infections, making alternative options necessary. Fosfomycin, an old antibiotic, is recommended for treating canine bacterial cystitis, especially in cases involving MDR infections where other treatments fail. This study analyzed 79 clinical *E. coli* isolates from dogs, determining their minimum inhibitory concentration (MIC) and mutant prevention concentration (MPC). Results indicated that 86.06% of the isolates were susceptible to fosfomycin. MIC_50_ and MIC_90_ were 4 mg/L and 96 mg/L, while MPC_50_ and MPC_90_ were 64 mg/L and 192 mg/L, respectively. These findings demonstrate that fosfomycin is highly effective against canine uropathogenic *E. coli*, including MDR strains. However, the high MPC values, particularly the MPC_90_, suggest the importance of susceptibility testing and ongoing resistance monitoring.

**Abstract:**

Fosfomycin is a bactericidal drug recommended as an alternative treatment for canine bacterial cystitis, particularly in cases involving multidrug-resistant (MDR) infections when no other options are available. In this study, minimum inhibitory concentration (MIC) and mutant prevention concentration (MPC) of fosfomycin were determined against 79 clinical *E. coli* isolates using the agar dilution method. The susceptibility rate of *E. coli* to fosfomycin was 86.06%, with MIC_50_ and MIC_90_ values of 4 mg/L and 96 mg/L, respectively. MPC_50_ and MPC_90_ values were 64 mg/L and 192 mg/L. Using pharmacokinetic (PK) data from dogs given a single 80 mg/kg oral dose of fosfomycin, the area under the curve per MIC_50_ (AUC_0–24_/MIC_50_) was 85.79 with time above MIC_50_ (T > MIC_50_) exceeding 50%. In urine, the AUC_0–24_/MIC_50_ was 10,694.78, and the AUC_0–24_/MPC_90_ was 222.81, with T > MPC_90_ extending beyond 24 h. Therefore, fosfomycin exhibited significant antibacterial activity against canine uropathogenic *E. coli*, including MDR strains, at concentrations below the susceptible MIC breakpoint. However, the high MPC values, especially the MPC_90_, indicate the critical importance of performing susceptibility testing for fosfomycin and maintaining ongoing resistance monitoring.

## 1. Introduction

Antimicrobial resistance is a significant challenge in clinical treatment. Inappropriate use of antibacterial drugs, such as misuse, overuse, or under-dosing, can contribute to this problem. In recent decades, the development of novel antibacterial agents has significantly slowed, even as the rise of resistant bacteria has accelerated, severely restricting treatment options. One strategy to address this issue is to revisit the older antibacterial drugs that have not been commonly used in clinical practice [1,2,3].

Urinary tract infections (UTIs) are one of the most prevalent infectious conditions in dogs. The primary bacterial pathogens responsible for canine UTIs include *Escherichia coli* (*E. coli*), *staphylococci*, *enterococci*, *Proteus* spp., and *Klebsiella* spp.; *streptococci*. *E. coli* is reported to cause approximately 46.7–63.6% of UTIs in dogs [4,5,6]. While beta-lactams and trimethoprim-sulfonamides are generally recommended as the first-line treatments for UTIs [7], some multidrug-resistant (MDR) *E. coli* strains are not susceptible to these nor the second-line drugs such as fluoroquinolones [8]. Last resort antibacterials like vancomycin or carbapenems are reserved for life-threatening infections. Additionally, the use of these last-resort drugs can be limited by factors such as resistance concerns, high expenses, or inconvenience regarding the administration routes in animals. Therefore, older drugs like fosfomycin might offer a viable alternative.

Fosfomycin, discovered in 1969, is an old bactericidal drug that inhibits cell wall biosynthesis and possesses a broad spectrum of activity. It is known for having minimal adverse effects and a high safety margin [9,10]. Resistance to fosfomycin typically involves a single mechanism, which does not usually lead to cross-resistance with other antibacterial drugs, making it less likely to contribute to multidrug resistance [11]. Fosfomycin-resistant mutants often exhibit slower growth and reduced adherence capabilities compared to wild-type bacterial strains [11]. Fosfomycin tromethamine, an oral formulation, has been approved by the Food and Drug Administration (FDA) for treating uncomplicated urinary tract infections caused by *E. coli* in humans since 1996 [12]. However, because of rising concerns about antimicrobial resistance and its significant impact on public health, fosfomycin has been designated as a critically important antimicrobial (CIA) by the World Health Organization (WHO) [13]. Additionally, it falls under Category A (avoid) according to the Antimicrobial Advice Ad Hoc Expert Group (AMEG) [14]. Currently, fosfomycin is not approved for veterinary use in the EU, and its administration to individual companion animals is only allowed under exceptional circumstances [14]. Despite these restrictions, fosfomycin is suggested as a treatment option for canine bacterial cystitis, particularly in cases of multidrug-resistant infections when alternative treatments are lacking [15,16]. Therefore, studying its antibacterial effectiveness could offer valuable insights into managing canine UTIs caused by *E. coli*.

The minimum inhibitory concentration (MIC) is a widely utilized measure for evaluating bacterial susceptibility to antimicrobial drugs. For MIC testing, a bacterial inoculum density of 10^5^ cfu/mL is typically used [17]. However, this inoculum density may not be sufficient to accurately determine susceptibility in certain cases [18]. Numerous infection cases have reported high bacterial loads in the urinary tract [19,20]. The mutant prevention concentration (MPC) represents the lowest antimicrobial concentration that prevents the growth of resistant subpopulations and is determined using a high bacterial density (≥10^9^ cfu/mL) [21,22]. Bacterial mutations occur at a natural frequency of approximately 10^6^–10^8^ cfu/mL, while bacterial infections can reach densities of up to 10^10^ cfu/mL [18]. Since MIC testing uses a bacterial density of 10^5^ cfu/mL, it may not account for spontaneous mutations. In contrast, MPC testing uses a high bacterial density (≥10^9^ cfu/mL), sufficient to include resistant mutants, and thus better represents bacterial burdens in severe infections [21,23,24]. While MIC testing inhibits susceptible bacteria, it does not affect the resistant subpopulation. MPC testing, however, inhibits both susceptible and resistant bacteria as single step of mutants.

This study aimed to explore the antibacterial efficacy of fosfomycin against clinical *E. coli* strains isolated from canine UTIs by assessing both the MIC and MPC values. The objective was to provide essential data for the efficient management of UTIs in dogs caused by *E. coli.*

## 2. Materials and Methods

### 2.1. Bacterial Identification

This study investigated 79 clinical isolates of *E. coli* obtained from dogs with naturally occurring UTIs. The dogs had been diagnosed at the Small Animal Hospital, Faculty of Veterinary Science, Chulalongkorn University, Bangkok, Thailand, from February 2017 to May 2018. These *E. coli* isolates had been previously identified by the Veterinary Diagnostic Laboratory, Faculty of Veterinary Science, Chulalongkorn University. The bacterial samples were grown on two types of agar plates, trypticase soy agar (TSA) with sheep blood and MacConkey agar, then incubated at 37 °C for 18–24 h. Colonies suspected of being Gram-negative bacteria underwent Gram staining. Further identification was conducted using an automated VITEK^®^ 2 system and Gram-negative identification cards following the manufacturer’s guidelines (BioMérieux, Marcy L’Étoile, France). Confirmed *E. coli* samples were preserved at −80 °C in a mixture of trypticase soy broth (TSB) and glycerol for future investigations.

### 2.2. Detection of Escherichia coli Strains Producing Extended-Spectrum Beta-Lactamase (ESBL)

The Extended-Spectrum Beta-Lactamase (ESBL) production of 79 clinical *E. coli* was assessed using a combination disk method, which involves comparing the size of inhibition zones around disks. Briefly, disks containing cefotaxime (30 μg) and ceftazidime (30 μg) (both third generation cephalosporins) were tested alone and in combination with clavulanic acid (30/10 μg), following the Clinical and Laboratory Standards Institute (CLSI) guidelines [17]. Each isolate was cultured on a TSA plate for 18–24 h at 37 °C. Then, bacterial colonies were suspended in 0.9% sodium chloride solution and adjusted to a turbidity corresponding to 0.5 McFarland standard using a densitometer (Biosan, Latvia). The prepared bacterial suspension was cultured onto a Mueller–Hinton agar (MHA) plate. Disks containing cefotaxime, ceftazidime, and their combinations with clavulanic acid were placed on the plate before being incubated for 18–24 h at 37 °C. Isolates were categorized as ESBL-producing if the zone of inhibition around a disk containing either cefotaxime or ceftazidime was at least 5 mm larger than the zone around the combination disk with clavulanic acid added [17].

### 2.3. Determination MIC of Other Antibacterial Drugs

The MICs for various antibacterial drugs were measured for all *E. coli* samples: ampicillin (2–32 mg/L), amoxicillin/clavulanic acid (2/1–32/16 mg/L), piperacillin (4–128 mg/L), cephalexin (4–64 mg/L), cefpodoxime (0.25–8 mg/L), cefovecin (0.5–8 mg/L), ceftiofur (1–8 mg/L), imipenem (1–16 mg/L), amikacin (2–64 mg/L), gentamicin (1–16 mg/L), tobramycin (1–16 mg/L), enrofloxacin (0.12–4 mg/L), marbofloxacin (0.5–4 mg/L), tetracycline (1–16 mg/L), nitrofurantoin (16–512 mg/L), chloramphenicol (2–64 mg/L), and sulfamethoxazole/trimethoprim (20 (1/19)–320 (16/304) mg/L). These measurements were obtained using the VITEK^®^ 2 system with Gram-negative Veterinary Susceptibility Test Cards (GN 65), following the manufacturer’s instructions (BioMérieux, Marcy L’Étoile, France). The MIC values for antibacterial drugs were interpreted based on their respective MIC breakpoints [25].

All seventy-nine clinical *E. coli* isolates were categorized into three types of antimicrobial resistance patterns based on their antibacterial resistance profiles: (1) non-multidrug resistance (NMDR, R < 3), which includes isolates resistant to fewer than three unrelated antibacterial classes; (2) multidrug resistance (MDR, R ≥ 3), which includes isolates resistant to at least one drug in three or more unrelated antibacterial classes; and (3) extreme-drug resistance (XDR, S ≤ 2), which includes isolates resistant to at least one drug in all but two or fewer antibacterial classes [26,27].

### 2.4. Determination MIC of Fosfomycin

The minimal inhibitory concentration (MIC) values were determined following the CLSI standard [17] using the agar dilution method. *E. coli* isolates were adjusted to a final concentration of approximately 1 × 10^5^ colony-forming units per spot and cultured on Mueller–Hinton agar (MHA) plates containing fosfomycin tromethamine (Sigma Chemical Co., Burlington, MA, USA) ranging from 0.125–256 mg/L, supplemented with 25 mg/L glucose-6-phosphate (Sigma-Aldrich, Taufkirchen, Germany). A 48-pin replicator (Sigma-Aldrich) was used to ensure uniformity, and the plates were then incubated for 18–24 h at 37 °C. All experiments were conducted in triplicate. The concentration of fosfomycin that prevented visible colony growth was noted, and interpretations were made based on MIC breakpoints: susceptible (S) if MIC was ≤64 mg/L, intermediate (I) if MIC was 128 mg/L, and resistant (R) if MIC was ≥256 mg/L, following CLSI guidelines [17]. *E. coli* ATCC 25,922 served as a quality control to ensure the accuracy of the testing process. The MIC results for all isolates were presented as a range, along with MIC_50_ (the concentration inhibiting 50% of total isolates) and MIC_90_ (the concentration inhibiting 90% of total isolates) values.

### 2.5. Determination MPC of Fosfomycin

Fosfomycin-susceptible *E. coli* isolates were selected for determining the mutant prevention concentration (MPC). To obtain a very large inoculum, each *E. coli* isolate was cultured on two MHA plates before being incubated at 37 °C for 18–24 h. Subsequently, bacterial colonies were cultured in Mueller–Hinton Broth (MHB) and incubated again at 37 °C for 18–24 h. The inoculum was estimated to have a concentration of 10^9^ cfu/mL using a spectrophotometer (Thermo Fisher Scientific, Agawam, MA, USA) with an absorbance reading of ≥1 at 540 nm [28]. The inoculum underwent centrifugation at 4000× *g* for 30 min at 4 °C, following which the supernatant was removed. The resulting pellets were then resuspended in a small volume of fresh cold MHB, adjusting the cell density to approximately >10^10^ cfu/mL. Viable counts of each inoculum were subsequently conducted using the serial dilution method to confirm a bacterial culture concentration of >10^10^ cfu/mL.

The inoculum, adjusted to a concentration of >10^10^ cfu/mL, was cultured on MHA plates containing various concentrations of fosfomycin (1, 2, 4, 8, 16, 32, 64, 96, 128, 192, and 256 mg/L), supplemented with 25 mg/L glucose-6-phosphate. The plates were then placed in a 37 °C incubator for 48 h, with visible growth checked every 24 h. MPCs were defined as the lowest concentration of fosfomycin that inhibited bacterial growth. To verify the MPC values, bacterial colonies from an MHA plate containing one concentration below the MPC were cultured onto MHA plates with fosfomycin at the MPC concentration and one concentration below the MPC. Each isolate underwent testing in triplicate, and *E. coli* ATCC 25,922 served as a quality control. The MPC range, MPC_50_, and MPC_90_ were calculated.

### 2.6. Estimation PK/PD of Fosfomycin

To evaluate the efficacy of fosfomycin, its pharmacokinetic-pharmacodynamic (PK/PD) properties were assessed using pharmacokinetic data from a previous study on dogs administered two different single oral doses: 40 mg/kg and 80 mg/kg [29]. For concentration-dependent drugs, efficacy is indicated by the AUC/MIC ratio (area under the drug concentration-time curve to the MIC) and the Cmax/MIC ratio (maximum drug concentration to the MIC). In contrast, for time-dependent drugs, efficacy is predicted by the T > MIC parameter, which represents the percentage of time during the dosing interval that drug concentrations exceed the MIC. For drugs exhibiting both concentration- and time-dependent killing, AUC/MIC or T > MIC parameters should be evaluated [30,31,32]. Typically, the Cmax/MIC ratio should be higher than 8–12, the AUC/MIC ratio should be higher than 125 for gram-negative bacteria, and the T > MIC should exceed 50% [33,34].

### 2.7. Statistical Analysis

Antibacterial susceptibility results were analyzed descriptively, with percentages of susceptibility and resistance reported at the 50th and 90th percentiles for MIC and MPC. Graphs were created using GraphPad Prism version 8 (GraphPad Software, San Diego, CA, USA) to visualize the data.

## 3. Results

### 3.1. Escherichia coli Isolates

All 79 clinical *E. coli* samples in our study were collected from dogs with UTIs. The ages of the subjects ranged from 1 to 16 years, with an average age of 7.8 years. Of these, 45.57% (36 out of 79) were female dogs and 54.43% (43 out of 79) were male dogs. However, the gender ratio provided is an additional detail and does not reflect prevalence rates of UTIs.

### 3.2. Escherichia coli Strains Producing Extended-Spectrum Beta-Lactamase (ESBL)

Twenty-five out of the seventy-nine *E. coli* isolates were confirmed as ESBL-producing through the double disk diffusion method. These findings corroborated the results from the VITEK^®^ 2 system, where bacterial samples were evaluated for their susceptibility to cefepime, cefotaxime, and ceftazidime (third generation cephalosporins), alone and in combination with clavulanic acid.

### 3.3. MIC of Other Antibacterial Drugs

The percentages of susceptibility and resistance are detailed in Table 1. The highest resistance rates were noted for ampicillin (94.74%), enrofloxacin (79.49%), and marbofloxacin (78.21%). In contrast, imipenem (94.87%), nitrofurantoin (91.03%), and amikacin (88.46%) exhibited the highest susceptibility rates.

Among the 25 ESBL-producing isolates, nitrofurantoin susceptibility was significantly lower compared to non-ESBL-producing *E. coli* (48% vs. 91.03%), while susceptibility to sulfamethoxazole/trimethoprim was similar between the two groups (48% in ESBL-producing *E. coli* vs. 47.44% in non-ESBL-producing *E. coli*). As expected, resistance to beta-lactams and fluoroquinolones was notably high at 96%.

The results indicated that 55.7% of all *E. coli* samples were MDR (*n* = 44/79) and 11.4% were XDR (*n* = 9/79). Among the 25 ESBL-producing isolates, 56% were MDR (*n* = 14/25) and 16% were XDR (*n* = 4/25). The antibacterial drugs most commonly associated with MDR and XDR were beta-lactams (including penicillins, penicillins with beta-lactamase inhibitors, and cephalosporins, excluding carbapenems), fluoroquinolones, and tetracyclines.

### 3.4. MIC of Fosfomycin

According to the CLSI guidelines, the susceptible breakpoint (S_bp_) for fosfomycin is ≤64 mg/L, and the resistant breakpoint (R_bp_) is ≥256 mg/L [17]. However, these MIC breakpoint values are established for *E. coli* urinary tract isolates in humans, with no data available for animals. Thus, the MIC interpretations in this study were based on these existing breakpoints.

**Figure 1:**
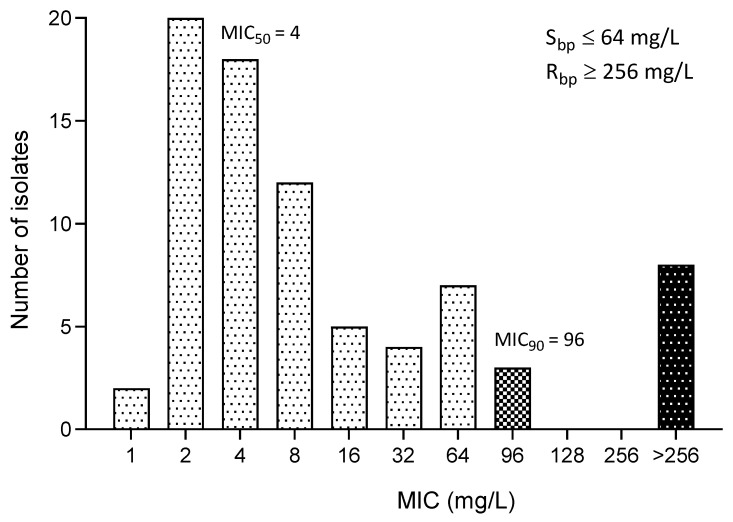
Distribution of fosfomycin MIC. Susceptible breakpoint, S_bp_; Resistant breakpoint, R_bp_. The white bar with black dot indicates susceptibility (MIC ≤ 64 mg/L). The grid bar indicates intermediate (MIC = >64 to <256 mg/L). The black bar with white dot indicates resistance (MIC ≥ 256 mg/L).

The MICs of fosfomycin for all 79 clinical *E. coli* isolates ranged from 1 to ≥256 mg/L. The distribution of MICs was presented in Figure 1. The most frequent MIC of fosfomycin was 2 mg/L (*n* = 20/79), while the MIC_50_ and MIC_90_ were 4 mg/L and 96 mg/L, respectively. Among all 79 *E. coli* samples from dogs with UTI, 86.06% (*n* = 68/79) were susceptible to fosfomycin as in Table 2. For the XDR *E. coli* isolates, 77.78% (*n* = 7/9) showed susceptibility to fosfomycin.

### 3.5. MPC of Fosfomycin

**Figure 2:**
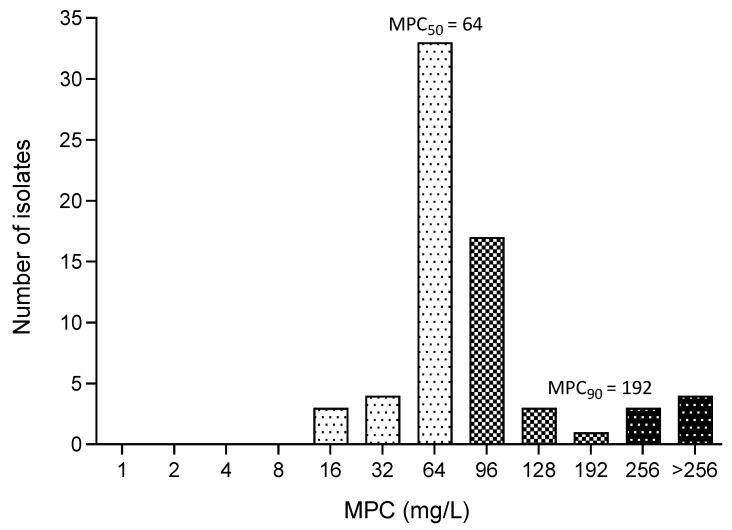
Distribution of fosfomycin MPC. The white bar with black dot indicates susceptibility (MIC ≤ 64 mg/L). The grid bar indicates intermediate (MIC = >64 to <256 mg/L). The black bar with white dot indicates resistance (MIC ≥ 256 mg/L).

To measure the MPC values, 68 fosfomycin-susceptible *E. coli* isolates (with MIC ≤ 64 mg/L) were selected. Since MPC breakpoints have not been established, susceptibility and resistance were determined based on MIC breakpoints. The distribution of MPC is shown in Figure 2, with the most frequent MPC for fosfomycin being 64 mg/L (*n* = 24/68). The MPC for all 68 fosfomycin-susceptible isolates ranged from 16 to ≥256 mg/L, as detailed in Table 3. Notably, 64.71% (*n* = 44/68) of these fosfomycin-susceptible isolates had an MPC ≤ 64 mg/L. Among the 22 fosfomycin-susceptible ESBL-producing *E. coli* samples, 50% (11/22) had an MPC ≤ 64 mg/L. The MPC for the 7 XDR *E. coli* isolates ranged from 48 to 256 mg/L, with MIC_50_ and MIC_90_ values at 96 and 256 mg/L, respectively. For all clinical *E. coli* isolates, the MPC_50_/MIC_50_ of fosfomycin was 16 (64/4), and the MPC_90_/MIC_90_ was 2 (192/96).

### 3.6. Estimation PK/PD of Fosfomycin

The pharmacokinetic parameters of fosfomycin in dogs were assessed using reference data from a previous study involving two different single oral doses: 40 mg/kg and 80 mg/kg [29]. The T > MIC values were derived from the drug concentration curve. At a dose of 40 mg/kg, fosfomycin achieved an AUC_0–24_/MIC_50_ of 36.37 in plasma (Table 4). Increasing the dose to 80 mg/kg resulted in an AUC_0–24_/MIC_50_ of 85.79 and a T > MIC_50_ greater than 12 h. In urine, the PK/PD ratios were significantly higher than in plasma due to the elevated concentration of fosfomycin in the urine (Table 5). Specifically, the AUC_0–24_/MIC or MPC in urine exceeded 125 for all MICs and the MPC_50_. Additionally, an 80 mg/kg dose of fosfomycin provided a T > MPC_90_ greater than 24 h.

## 4. Discussion

In this study, the susceptibility of UTI pathogens to first-line drugs was evaluated. Both amoxicillin-clavulanic acid and sulfamethoxazole/trimethoprim had a susceptibility rate of 47.44%. Fluoroquinolones, specifically enrofloxacin and marbofloxacin, exhibited low susceptibility rates of 15.38% and 20.31%, respectively. Fortunately, the bacteria showed high susceptibility to less commonly used drugs such as imipenem (94.87%), nitrofurantoin (91.03%), and amikacin (88.46%). The low susceptibility rates for fluoroquinolones may be attributed to their frequent use in Thailand [35]. In contrast, the high susceptibility rates to alternative drugs like nitrofurantoin and amikacin may result from their infrequent use due to significant side effects [15] and limited accessibility in Thailand.

The MICs of fosfomycin were determined against clinical *E. coli* isolates from dogs with UTIs. The MIC values ranged from 1.0 to >256 mg/L, with MIC_50_ and MIC_90_ at 4 and 96 mg/L, respectively. In humans, the susceptible breakpoint for fosfomycin was ≤64 mg/L, and the resistant breakpoint was ≥256 mg/L [17]. However, MIC breakpoints for animals have not yet been established. Therefore, this study used human CLSI guidelines to assess bacterial susceptibility to fosfomycin. Among all *E. coli* samples, 86.06% (*n* = 68/79) were susceptible to fosfomycin, with an MIC_50_ of 4 mg/L. For multidrug-resistant (MDR) *E. coli* (*n* = 44/79), 79.55% (35/44) were susceptible to fosfomycin. These findings indicate high susceptibility rates of clinical UTI bacteria, including MDR isolates, to fosfomycin.

Previous studies support these results, such as that of Hubka and Boothe (2011), who reported MICs of fosfomycin for canine UTI isolates in the United States (*n* = 200, 2008–2010) ranging from 0.25 to 196 mg/L, with a susceptibility rate of 98.9%. Among MDR isolates, 54% (*n* = 108/200) were susceptible to fosfomycin, with MIC_50_ and MIC_90_ at 1 and 3 µg/mL, respectively [36].

In this study, the MIC_50_ for all samples, including MDR *E. coli*, was 4 mg/L, while the MIC_90_ for MDR *E. coli* reached 256 mg/L, the fosfomycin resistance breakpoint. The broad MIC range and high MIC_90_ indicate potential fosfomycin resistance, making susceptibility testing essential, particularly for MDR bacteria. Fosfomycin is a promising alternative for UTI treatment, but resistance monitoring is crucial. Previous research supports these findings, showing that bactericidal effects occurred only at high (plasma) fosfomycin concentrations (32–64 mg/L). After 24 h, bacterial regrowth was observed when (plasma) fosfomycin levels were at 0.5–32 mg/L but not at 64 mg/L, likely due to spontaneous mutants requiring higher concentrations than the MIC. Thus, continuous monitoring of UTI *E. coli* sensitivity to fosfomycin is necessary [37]. Another study suggests that oral fosfomycin trometamol could be a viable step-down therapy for patients with MDR *E. coli* urinary tract infections, but a higher relapse rate was observed [38].

To determine the MPC values, 68 *E. coli* samples with MICs at or below the susceptible breakpoint (MIC ≤ 64 mg/L) were selected. Among these, 41.18% (*n* = 28) had MPCs exceeding the susceptible breakpoint. Although these samples initially showed high susceptibility to fosfomycin based on low MIC values, an increased bacterial population allowed mutations to occur, leading to heteroresistance and the emergence of resistant mutants under selective pressure from antibacterial drugs.

A previous study reported MPC values for fosfomycin in *E. coli* heteroresistant or resistant subpopulations to be greater than 1024 mg/L, with MICs ranging from 4 to 32 mg/L [39]. Another study found MPC of fosfomycin in wild type *E. coli* (*E. coli* ATCC 25,922) to be 57.6 mg/L, with MICs ranging from 1 to 2 mg/L [40]. The high MPC, particularly the MPC_90_ observed in our study, suggests that susceptibility testing for fosfomycin is essential and that ongoing resistance monitoring may be necessary. However, a low correlation between MIC and MPC in clinical *E. coli* with various antibacterial drugs (R^2^ < 0.3) has been reported [41]. MPC can vary widely due to factors such as bacterial strains, antibacterial drugs, bacterial density, and mutation types. Even replicates of the same strain with the same drug can yield different MPC results. Moreover, the MPC of antibacterial-resistant strains is typically higher than that of wild-type strains [42]. Therefore, MPC should be measured individually, and high variability should be considered when using MPC to inhibit mutant subpopulations.

Using an antibacterial drug at a dosage that achieves plasma concentrations equal to or above the MPC can theoretically prevent the growth of mutant subpopulations. However, for effective resistance prevention in clinical treatments, it is crucial to maintain high drug concentrations at the infection sites. Therefore, pharmacokinetics should be evaluated alongside MPC. Additionally, several factors need to be considered, including the host’s immune response for pathogen clearance, potential adverse effects from high drug dosages, the health status, and the cost of the medication.

Three PK/PD parameters (AUC_0–24_/MIC, Cmax/MIC, and T > MIC) are used to assess bacterial killing properties. Fosfomycin, however, does not fit neatly into the categories of concentration-dependent or time-dependent drugs. According to Falagas et al. (2016), fosfomycin’s bacterial killing properties may vary depending on the pathogen [43]. Fosfomycin exhibits time-dependent properties against *Pseudomonas aeruginosa* and *Staphylococcus aureus* [44,45], while it demonstrates concentration-dependent properties against *Enterococcus faecium*, *Proteus mirabilis*, and *E. coli* [46,47]. Additionally, fosfomycin has been suggested to possess both concentration- and time-dependent characteristics in *S. aureus* [48]. Moreover, fosfomycin’s efficacy against *E. coli* was best estimated by AUC/MIC, given the strong correlation between in vivo efficacy and AUC/MIC (R^2^ = 0.9227) [49].

Previous research conducted in murine models investigated the efficacy of fosfomycin against *E. coli* isolates. A static dose was observed when AUC_0–24_/MIC ratios ranged from 8.5 to 49.4 (mean = 23.7), with T > MIC ranging from 15 to 68% (mean = 38.6%). For studies achieving a one-log kill (bactericidal) dose, observed AUC/MIC ratios ranged from 28 to 193 (mean = 98.9), with T > MIC ranging from 52 to 100% (mean = 75.8%) [50]. Target values for fosfomycin’s clinical efficacy (static dose) should be approximately >23.7 for AUC_0–24_/MIC ratios and >38.6% for T > MIC. For achieving a one-log kill (bactericidal) dose, target values should be approximately > 98.9 for AUC_0–24_/MIC ratios and >75.8% for T > MIC.

In another study, an in vitro investigation examined fosfomycin’s activity against ESBL-producing *E. coli* isolates, representing antibacterial-resistant mutants. Bactericidal activity and resistance suppression were observed when AUC_0–24_/MIC was 3136, with T > MIC maintained throughout the experiment [51]. Target values for fosfomycin’s efficacy against antibacterial-resistant mutants should be approximately >3136 for AUC_0–24_/MIC ratios.

In plasma, fosfomycin treatment at doses of 40 and 80 mg/kg resulted in AUC_0–24_/MIC_50_ values of 36.37 and 85.79, respectively. According to target values from a previous study (AUC_0–24_/MIC_50_ ≥ 23.7) [50], these doses achieved optimal plasma concentrations for inhibiting bacterial growth when the MIC was ≤4 mg/L (MIC_50_). However, neither dose showed antibacterial activity in plasma when the fosfomycin MIC was ≥96 mg/L (MIC_90_).

In urine, all PK/PD ratios were significantly greater than those in plasma due to the high concentration of fosfomycin in urine [29]. According to the target values for bactericidal concentration (one-log kill dose) from the previous study (AUC_0–24_/MIC_50_ ≥ 98.9) [50], administration of fosfomycin at either 40 or 80 mg/kg resulted in PK/PD parameters reaching bactericidal levels in urine when the bacterial MIC was ≤192 mg/L (MPC_90_). Additionally, fosfomycin at either dosage provided AUC_0–24_/MIC_50_ ratios sufficient to suppress resistance, based on the target values for antibacterial-resistant mutants (AUC_0–24_/MIC ≥ 3136) [51]. The PK/PD results from this study indicated that fosfomycin effectively inhibits canine UTI *E. coli*, particularly in urine. However, these PK/PD ratios are based on previous findings, and clinical outcomes may vary due to factors such as individual health status, host immunity, bacterial burden, and environment.

## 5. Conclusions

Clinical *E. coli* samples from dogs with UTIs showed a high susceptibility rate to fosfomycin at 86.06%, with MIC_50_ and MIC_90_ values of 4 mg/L and 96 mg/L, respectively. The MPC ranged from 16 to ≥256 mg/L, with MPC_50_ and MPC_90_ at 96 mg/L and 192 mg/L, respectively. The MPC_50_/MIC_50_ ratio was 16, while the MPC_90_/MIC_90_ ratio was 2. Fosfomycin demonstrated significant antibacterial activity against canine uropathogenic *E. coli*, including MDR strains, at concentrations below the susceptible MIC breakpoint. However, the high MPC values, especially the MPC_90_, indicate the importance of conducting susceptibility testing for fosfomycin and the need for continuous resistance monitoring.

## Figures and Tables

**Table 1:** Antibacterial susceptibility of UTI *E. coli* isolates detected by VITEK^®^ 2 system.

Antibacterial Drugs	%S	%I	%R
Ampicillin	5.26	NA	94.74
Amoxicillin	47.44	12.82	39.74
Piperacillin	24.36	2.56	73.08
Cephalexin	28.21	7.69	64.10
Cefpodoxime	50.00	NA	50.00
Cefovecin	53.85	3.85	42.31
Ceftiofur	50.00	2.56	47.44
Imipenem	94.87	1.28	3.85
Amikacin	88.46	NA	11.54
Gentamicin	56.41	2.56	41.03
Tobramycin	55.13	30.77	14.10
Enrofloxacin	15.38	5.13	79.49
Marbofloxacin	20.51	1.28	78.21
Tetracycline	30.77	2.56	66.67
Nitrofurantoin	91.03	8.97	3.85
Chloramphenicol	50.00	20.51	29.49
Sulfamethoxazole/trimethoprim	47.44	NA	52.56

NA = not applicable, S = susceptible, I = intermediate, and R = resistant.

**Table 2:** MICs of fosfomycin.

MIC Parameters	All *E. coli*	ESBL-Producing *E. coli*	MDR *E. coli*
	(*n* = 79)	(*n* = 25)	(*n* = 44)
Range of MIC (mg/L)	1—≥256	2—≥256	2—≥256
MIC_50_ (mg/L)	4	4	4
MIC_90_ (mg/L)	96	96	256
Susceptibility (%)	86.06	88.89	79.55

**Table 3:** MPC and MPC/MIC of fosfomycin.

MPC Parameters	All *E. coli*	ESBL-Producing *E. coli*	MDR *E. coli*
	(*n* = 68)	(*n* = 22)	(*n* = 35)
Range of MPC (mg/L)	16—≥256	48—≥256	16—≥256
MPC_50_ (mg/L)	64	64	64
MPC_90_ (mg/L)	192	192	256
MPC_50_/MIC_50_	16	16	16
MPC_90_/MIC_90_	2	2	1

**Table 4:** PK/PD of fosfomycin with canine plasma.

PK/PD	Parameters	40 mg/kg PO	80 mg/kg PO
PK (plasma) [29]	AUC_0–24_ (mg*h/L)	145.47	343.16
	Cmax (mg/L)	34.46	66.40
	T (h)	24	24
PD	MIC_50_ (mg/L)	4	
	MIC_90_ (mg/L)	96	
	MPC_50_ (mg/L)	64	
	MPC_90_ (mg/L)	192	
PK/PD (MIC_50_)	AUC/MIC_50_	36.37	85.79
	Cmax/MIC_50_	8.62	16.60
	T > MIC_50_	<30%	>50%
PK/PD (MIC_90_)	AUC/MIC_90_	1.52	3.57
	Cmax/MIC_90_	0.36	0.69
	T > MIC_90_	0%	0%
PK/PD (MPC_50_)	AUC/MPC_50_	2.27	5.36
	Cmax/MPC_50_	0.54	1.04
	T > MPC_50_	0%	<10%
PK/PD (MPC_90_)	AUC/MPC_90_	0.76	1.79
	Cmax/MPC_90_	0.18	0.35
	T > MPC_90_	0%	0%

PO = per oral; AUC_0–24_ = the area under drug concentration–time curve from time zero to the time 24 h; Cmax = maximum concentration; T = time 24 h.

**Table 5:** PK/PD of fosfomycin with canine urine.

PK/PD	Parameters	40 mg/kg PO	80 mg/kg PO
PK (urine) [29]	AUC_0–24_ (mg × h/L)	15,390.22	42,779.13
	Cmax (mg/L)	4463.07	8784.93
	T (h)	24	24
PD	MIC_50_ (mg/L)	4	
	MIC_90_ (mg/L)	96	
	MPC_50_ (mg/L)	64	
	MPC_90_ (mg/L)	192	
PK/PD (MIC_50_)	AUC/MIC_50_	3847.56	10,694.78
	Cmax/MIC_50_	1115.77	2196.23
	T > MIC_50_	100%	100%
PK/PD (MIC_90_)	AUC/MIC_90_	160.31	445.62
	Cmax/MIC_90_	46.49	91.51
	T > MIC_90_	>50%	100%
PK/PD (MPC_50_)	AUC/MPC_50_	240.47	668.42
	Cmax/MPC_50_	69.74	137.26
	T > MPC_50_	>50%	100%
PK/PD (MPC_90_)	AUC/MPC_90_	80.16	222.81
	Cmax/MPC_90_	23.25	45.75
	T > MPC_90_	<50%	100%

PO = per oral; AUC_0–24_ = the area under drug concentration–time curve from time zero to the time 24 h; Cmax = maximum concentration; T = time 24 h.

## Data Availability

The data that support the findings of this study are available from the corresponding author upon reasonable request.
